# Iatrogenic cushing’s syndrome post intralesional triamcinolone acetonide in oral submucous fibrosis: 2 case reports

**DOI:** 10.1186/s12903-023-03505-x

**Published:** 2023-10-28

**Authors:** Kuenga Choden, Tshewang Gyeltshen

**Affiliations:** 1 Department of Dentistry, Tsirang Hospital, Tsirang, Bhutan; 2 Graduate School of Public Health, St. Luke’s International University, Tokyo, Japan

**Keywords:** Iatrogenic cushing syndrome, Triamcinolone acetonide injection, Oral submucous fibrosis, Betel nut chewing, Oral potentially malignant disorders

## Abstract

**Background:**

Oral Submucous Fibrosis (OFMF) is an oral potentially malignant disorder (OPMDs), strongly linked to betel quid chewing. It exhibits a significantly higher rate of malignant transformation compared to other OPMDs. The use of Intralesional Triamcinolone Acetonide Injection has emerged as a highly effective treatment option and has become the cornerstone of managing this condition.

**Case Presentation:**

A 44-year-old female and a 40-year-old male presented with burning sensation and limited mouth opening, leading to diagnosis of OSMF. Both patients were treated with Triamcinolone Acetonide (TAC) Intralesional injections. Following a few months of treatment, a significant improvement in mouth opening was observed. However, both patients began experiencing symptoms such as facial rounding (mooning of the face), a buffalo hump, uneven hair growth, and swelling in the lower extremities. Upon recognizing these symptoms as indicative of Cushing’s Syndrome, the administration of TAC injection was discontinued. Both patients were referred to a higher-level medical facility for confirmatory tests, which revealed elevated cortisol levels in both morning (Cortisol A.M) and evening (Cortisol P.M).

**Conclusion:**

TAC injection has been established as an effective treatment for OSMF. However, it is crucial to closely monitor patients for any adverse effects resulting from the treatment, which may arise from high dosage or increased frequency.

## Introduction

Oral Submucous Fibrosis (OSMF) is defined by the World Health Organization (WHO) as a slowly progressive disease characterized by the formation of fibrous bands in the oral mucosa. These bands eventually cause a significant limitation in mouth movement, including the movement of the tongue [1]. OSMF is commonly characterized by chronic inflammation and abnormal collagen deposition in the oral mucosa. This oral potentially malignant disorder (OPMDs) manifests with various symptoms, including ulceration, xerostomia (dry mouth), a burning sensation, and limitation of mouth opening. The extent of sites involved, and its rigidity usually depicts the severity of OSMF. These symptoms significantly impact the quality of life for individuals affected by the condition.

Earlier reports have indicated that OSMF has a high malignant transformation rate, which ranges from 1.9 to 9% [3]. The condition is highly prevalent among the population where areca-nut chewing is common. Moreover, a significant number of OSMF patients who chewed areca nut were found to have a concurrent issue with alcoholism and other drugs putting them at risk of other co-morbidities. In addition, metabolic syndromes such as hyperglycemia, hypertension, dyslipidemia, and abnormal obesity were significantly associated with areca nut chewers [3].

Cushing’s syndrome is a condition that develops due to prolonged exposure to glucocorticoids, leading to a constellation of associated signs and symptoms. These include facial rounding (moon face), changes in hair distribution, the development a buffalo hump at the base of the neck, a dusky plethoric appearance with the formation of purple stretch marks (striae), muscle weakness, high blood pressure (vascular hypertension), increased sugar in the urine (glycosuria), and the presence of protein in the urine (albuminuria) [4].

We present two cases of Iatrogenic Cushing’s Syndrome that developed in patients with OSMF following treatment with Triamcinolone Acetonide (TAC) intralesional injection.

### Case 1

A 44-year-old female presented at a dental clinic with complaints of limited mouth opening, burning sensation when consuming spicy foods, and pain upon mouth opening. The patient reported a 20-year history of betel quid chewing and a more recent habit of consuming commercially available areca products called “Sakila Supari” (betel nut mixed with artificial sweeteners readily available in the market) for the past 5 years. At the time of the presentation, she had not discontinued her habit.

During intra-oral examination, bilateral blanching of the buccal mucosa extending to the pterygomandibular raphe region was observed. The lips showed signs of tightness against the teeth, suggesting their involvement. The inter-incisal width was restricted to 15 mm. Extensive staining was evident on the tooth surfaces. Upon palpation, it was observed that the buccal mucosa exhibited a firm, rubbery, and non-elastic texture. The assessment indicated that the involvement did not extend into the oropharynx. The patient had unremarkable medical history and was not currently on any medications (as shown in Table 1).

**Table 1:** Patients Personal and Medical History at the time of presentation, Case 1

Personal History		Medical History	
Age	44	Blood Pressure	130/86
Sex	Female	Anemia	No
Occupation	Farmer	Icterus	No
Tobacco Use (Smoked/non-smoked)	No	Edema	No
Areca nut (Betel quid) chewing	Yes	Co-morbidities	No
Commercially processed areca products	Yes	Current Medications	No
Alcohol	No	Past Medical History	None

### Diagnosis

Based on the clinical features and personal history, the patient was provisionally diagnosed as having advanced OSMF classified under stage 3 of clinical staging and M4 of functional stagging (S3M4) as per the classification proposed by More, et al. [5]. While histopathological evaluation would have aided the diagnosis, we were not able to perform the test due to unavailability of histopathological tests in the hospital.

### Treatment & management

Initially, 2 ml intralesional injections of Placentrex were administered twice a week for 6 months, but no improvement was observed in the patient’s condition. Consequently, Placentrex treatment was discontinued, and 1 ml Triamcinolone Acetonide (TAC) once weekly was chosen as an alternative for OSMF treatment. TAC was administered via intralesional injections once a week, and the patient was advised to perform regular mouth opening exercises. Both intralesional injections were performed using standard dental needle by emptying the dental cartridge first and filling the TAC/Placentrex in the dental cartridge. No local anesthetics were used in combination or alone in the treatment. Additionally, oral vitamin supplements, vitamin C (250 mg twice daily) and vitamin B complex (1 tablet, twice daily) were prescribed. Notably, there was a significant improvement in mouth opening after 4 months of treatment initiation.

**Fig. 1:**
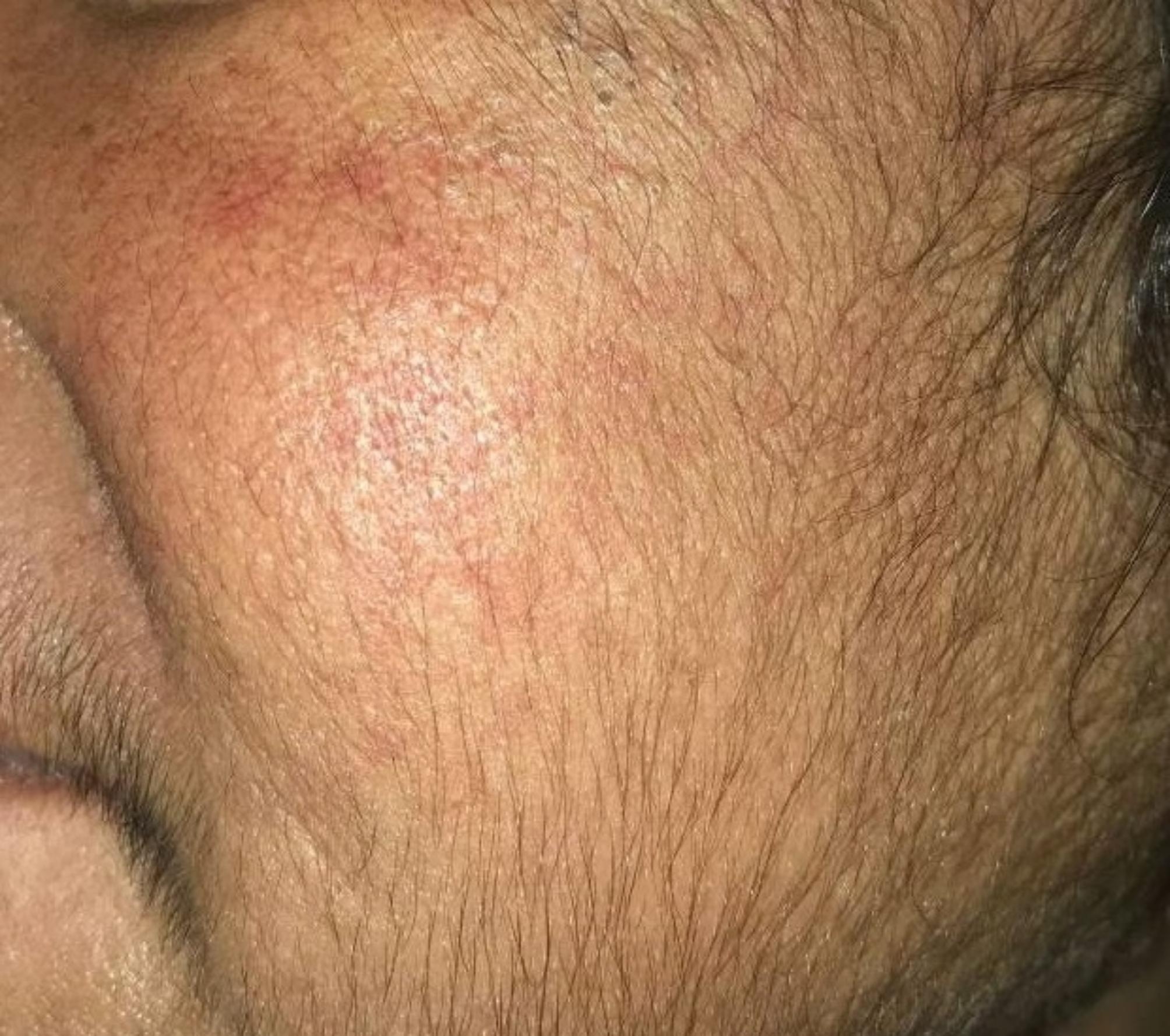
Facial Striae and Hirsutism

**Fig. 2:**
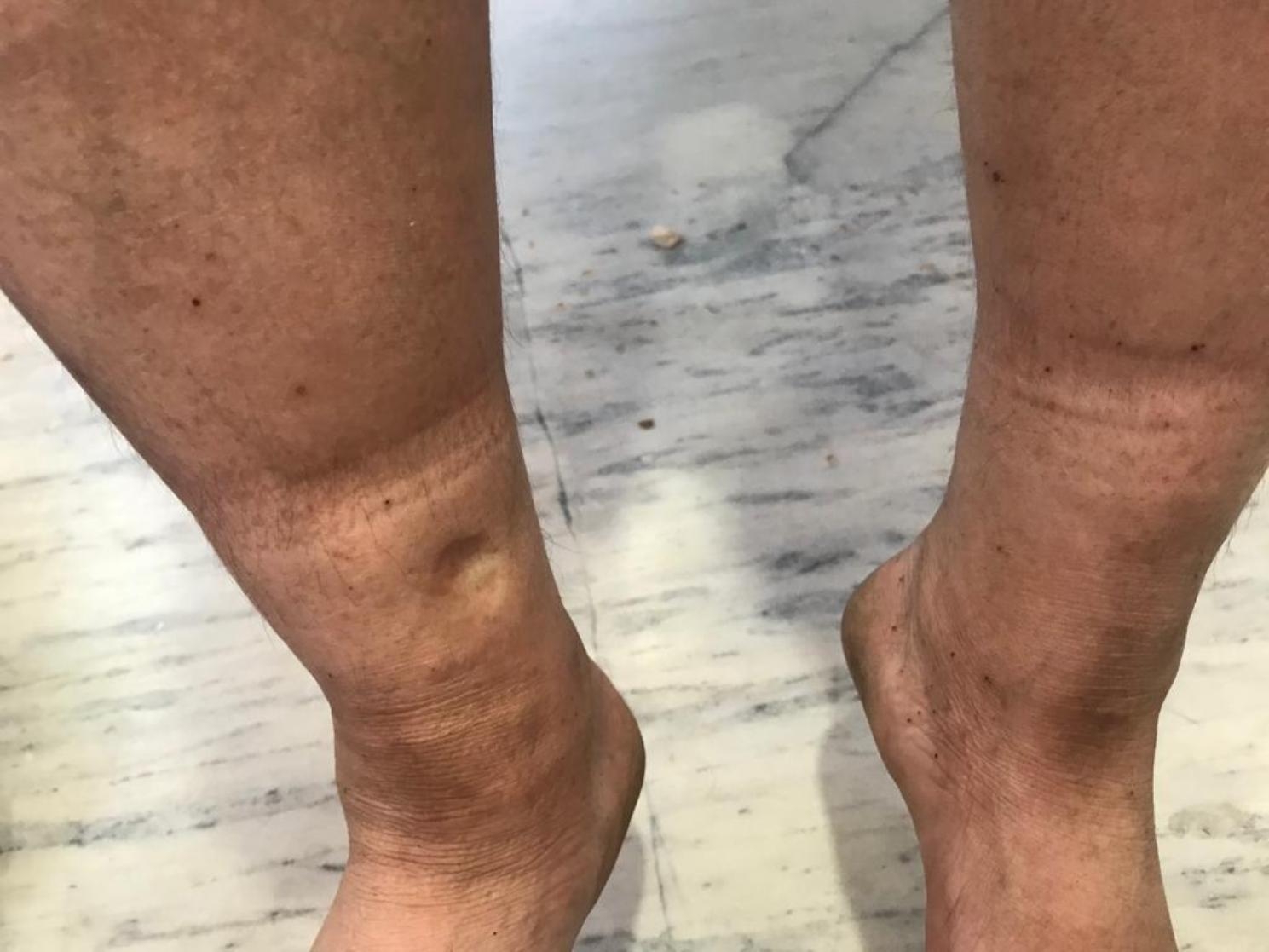
Bilateral Pedal Edema

**Fig. 3:**
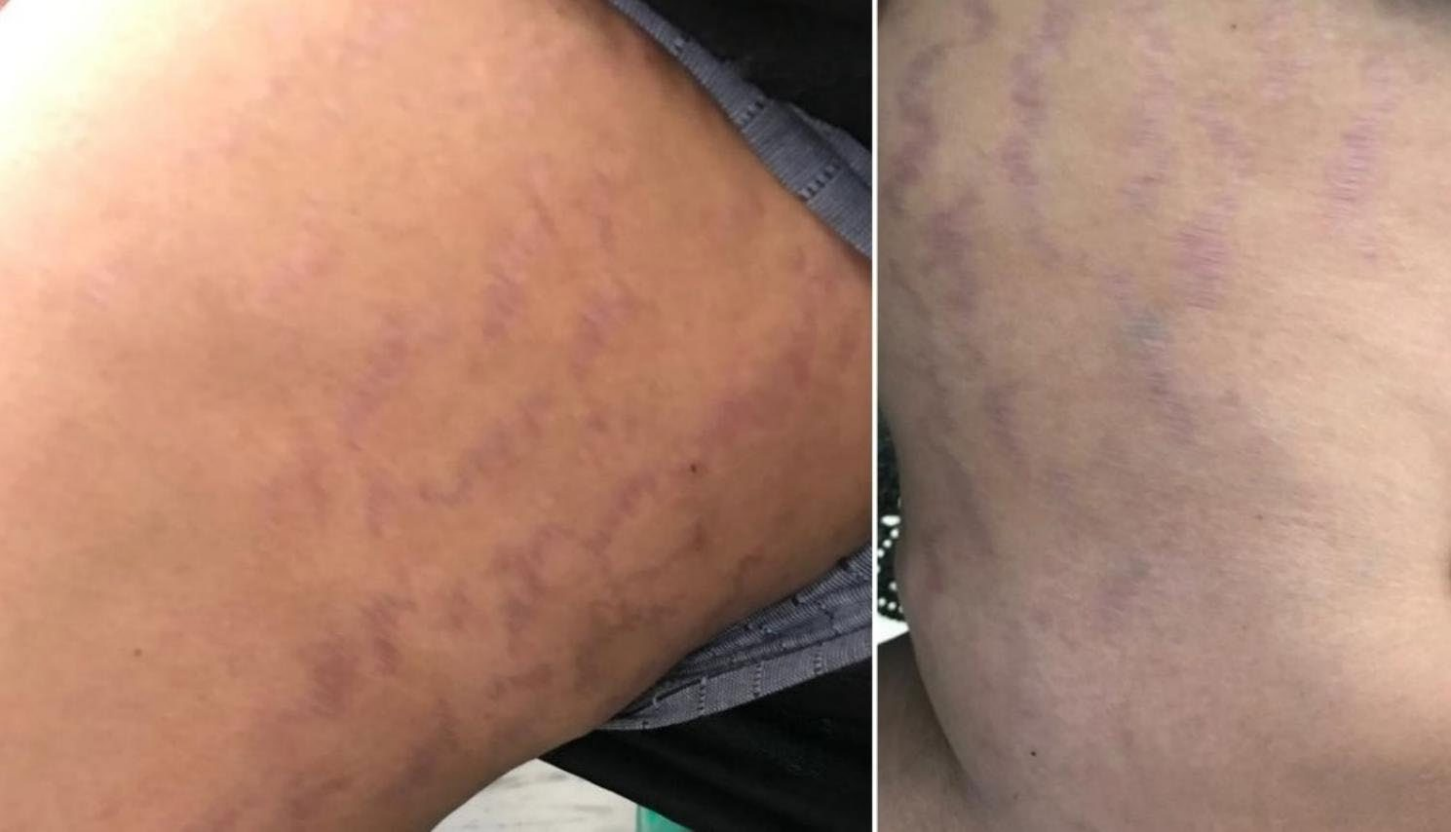
Purple Striae of thighs

However, the patient later presented with symptoms consistent with Cushing’s syndrome. These included a moon-like rounding of the face, increased facial and bodily hair growth (Fig. 1), the characteristics “moon face” appearance, and noticeable purple striae in the lower extremities (Fig. 2). Additionally, bilateral pedal edema (Fig. 3), along with raised blood pressure and weight gain – typical of Cushing’s syndrome, were evident. The patient also reported experiencing oligomenorrhea.

Upon recognizing the symptoms of Cushing’s syndrome, Triamcinolone Acetonide treatment was promptly discontinued, and the patient was referred to a physician for further confirmatory tests. These tests confirmed elevated cortisol levels (both cortisol A.M and cortisol P.M). The patient was scheduled for regular reviews every two months. After one year, it was observed that the symptoms of Cushing’s syndrome had resolved, and both morning (Cortisol A.M) and evening (Cortisol P.M) cortisol levels were found to be within the normal ranges.

## Case 2

**Fig. 4:**
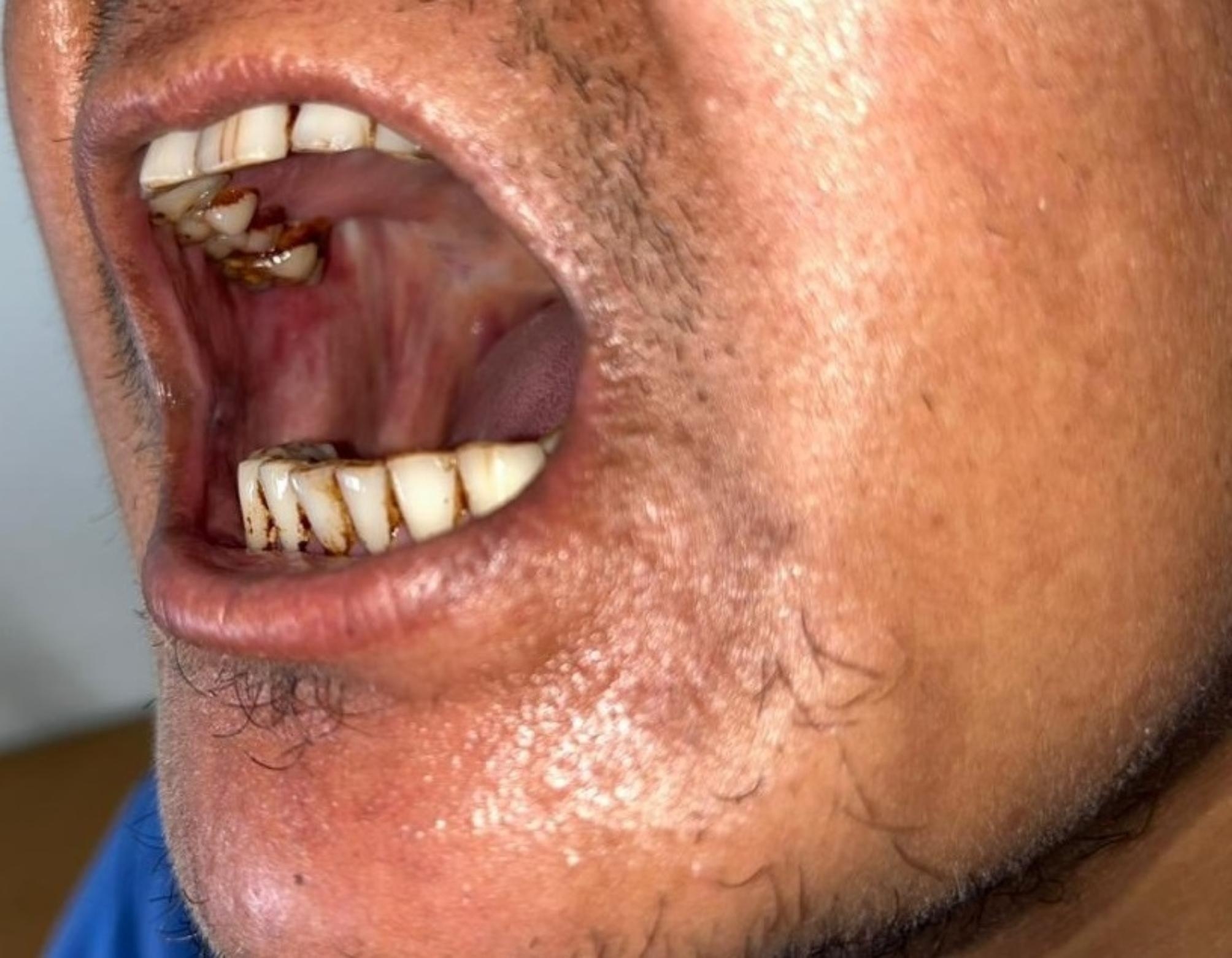
Improved Mouth Opening after the TAC injection

A 40-year-old male presented with limited mouth opening and burning sensation of the buccal mucosa. Upon examination, the mouth opening of 20 mm interincisal width was observed with palpable fibrous band over the bilateral buccal mucosa extending to the oropharynx. Subsequently, the patient was diagnosed as having Oral Submucous Fibrosis (OSMF) under stage 2 of clinical stagging and M3 of functional stagging (S2M3) [5]. The patient had unremarkable medical history (Table 2) and received treatment with intralesional TAC injections (1ml) twice a week. After 2 months and 15 days of treatment, the patient had remarkable improvement of mouth opening (Fig. 4). However, the patient presented visible facial swelling, hair loss in the lower extremities, the formation of a buffalo hump at the base of the neck, elevated blood pressure, and notable weight gain. These symptoms strongly suggested the development of Cushing’s syndrome likely due to the TAC injection.

Based on the clinical presentation, the patient was suspected to have developed Cushing’s syndrome. Immediate discontinuation of Triamcinolone Acetonide (TAC) usage was initiated, and the patient was promptly referred to a physician for further management and evaluation. Based on the clinical symptoms, the patient was diagnosed with Iatrogenic Cushing syndrome by the treating physician.

**Table 2:** Patients Personal and Medical History at the time of presentation, Case 2

Personal History		Medical History	
Age	40	Blood Pressure	128/85
Sex	Male	Anemia	No
Occupation	Civil Servant	Icterus	No
Tobacco Use (Smoked/non-smoked)	Yes	Edema	No
Areca nut (Betel quid) chewing	Yes	Co-morbidities	No
Commercially processed areca products	Yes	Current Medications	No
Alcohol	Yes	Past Medical History	None

## Discussion

Studies conducted in Bhutan have reported that nearly half of the Bhutanese population consumes betel quid [6]. Betel quid is a preparation that typically includes ingredients such as areca nut, betel leaf, slaked lime, and sometimes tobacco. The widespread consumption of betel quid in Bhutan exposes a significant portion of the population to the potential health risks associated with its use, including the development of oral health conditions like OSMF and an increased risk of oral cancer. In 2004, the International Agency for Research on Cancer (IARC) classified areca nut and betel quid as a ‘Group 1 Carcinogen’ [7].

While the diagnosis of OSMF is relies on clinical and histopathological evaluation, treatment approaches for OSMF vary widely in the literature, and there is currently no universally recognized effective treatment [8]. Intralesional corticosteroid injections, designed to mitigate epithelial lining fibrosis and enhance mouth opening through enzymatic actions, represent viable treatment alternatives for early-stage cases that do not necessitate surgical intervention [9]. With a current prevalence rate of 4.96%, OSMF stands as a prominent condition among the Oral Mucosal Potentially Malignant Disorders (OMPDs). Notably, the primary risk factor attributed to the onset of OSMF is the consumption of Areca nut and its commercially available products.

A combination of drugs, including steroids, hyaluronidase, human placenta extracts, chymotrypsin, and collagenase, as well as pentoxifylline, nylidrin hydrochloride, iron, and multivitamin supplements, including lycopene, have been utilized in the treatment of OSMF. In a three-year follow-up study involving 400 participants, lycopene has emerged as an effective and viable treatment option for OSMF [10]. Another systematic review reports lycopene to be safe and effective treatment option for the OSMF [11]. Steroids play a crucial role in preventing or suppressing inflammatory reactions, thereby reducing fibrosis by decreasing fibroblastic proliferation and collagen deposition. Triamcinolone Acetonide (TAC) intralesional injections have also demonstrated their effectiveness in OSMF treatment, leading to notable improvements in mouth opening [12]. This use of TAC injection in combination with others is shown to be highly effective in a meta-analysis reported by Gopinath et al. [13].

While these treatment options have been reported to be effective, it is important to be aware that TAC, being four times more potent than hydrocortisone, can elevate systemic cortisol levels and potentially lead to Hypercortisolism. Such cases have been reported earlier in various oral and non-oral use of corticosteroids [14]. TAC is a fluorinated prednisolone derivative with lower solubility compared to the parent compound, which allows it to remain at the injection site for an extended period. This slow release of steroids can potentially elevate systemic steroid levels, potentially causing Cushing’s Syndrome.

## Conclusion

In conclusion, Oral submucous fibrosis (OSMF) is a prevalent disease, particularly in populations where areca nut consumption is common. Many patients do not notice the early symptoms of OSMF, leading to delayed diagnosis and presentation at advanced stages with restricted mouth opening. OSMF is an OOMDs that can progress to malignancy if left untreated.

Triamcinolone Acetonide has been documented as an effective treatment for OSMF. Nevertheless, it is imperative to maintain vigilant monitoring of patients for potential adverse effects that may stem from high dosages or increased frequency of administration. Clinicians should remain watchful and attentive to any changes noted by patients throughout the treatment regimen. Early detection, timely intervention, and meticulous monitoring are of paramount importance in managing OSMF, as they can help prevent disease progression and mitigate the risk of potential complications.

## Data Availability

The photographic data used in this report are available from the corresponding author upon reasonable request.
